# Diagnostic accuracy of magnetic resonance imaging techniques for treatment response evaluation in patients with head and neck tumors, a systematic review and meta-analysis

**DOI:** 10.1371/journal.pone.0177986

**Published:** 2017-05-24

**Authors:** Anouk van der Hoorn, Peter Jan van Laar, Gea A. Holtman, Henriette E. Westerlaan

**Affiliations:** 1 University of Groningen, University Medical Center Groningen, Department of Radiology, Groningen, The Netherlands; 2 University of Groningen, University Medical Center Groningen, Medical Imaging Center, Groningen, The Netherlands; 3 University of Groningen, University Medical Center Groningen, Department of General Practice, Groningen, The Netherlands; Universita degli Studi di Firenze, ITALY

## Abstract

**Background:**

Novel advanced MRI techniques are investigated in patients treated for head and neck tumors as conventional anatomical MRI is unreliable to differentiate tumor from treatment related imaging changes.

**Purpose:**

As the diagnostic accuracy of MRI techniques to detect tumor residual or recurrence during or after treatment is variable reported in the literature, we performed a systematic meta-analysis.

**Data sources:**

Pubmed, EMBASE and Web of Science were searched from their first record to September 23^th^ 2014.

**Study selection:**

Studies reporting diagnostic accuracy of anatomical, ADC, perfusion or spectroscopy to identify tumor response confirmed by histology or follow-up in treated patients for head and neck tumors were selected by two authors independently.

**Data analysis:**

Two authors independently performed data extraction including true positives, false positives, true negatives, false negatives and general study characteristics. Meta-analysis was performed using bivariate random effect models when ≥5 studies per test were included.

**Data synthesis:**

We identified 16 relevant studies with anatomical MRI and ADC. No perfusion or spectroscopy studies were identified. Pooled analysis of anatomical MRI of the primary site (11 studies, *N* = 854) displayed a sensitivity of 84% (95%CI 72–92) and specificity of 82% (71–89). ADC of the primary site (6 studies, *N* = 287) showed a pooled sensitivity of 89% (74–96) and specificity of 86% (69–94).

**Limitations:**

Main limitation are the low, but comparable quality of the included studies and the variability between the studies.

**Conclusions:**

The higher diagnostic accuracy of ADC values over anatomical MRI for the primary tumor location emphases the relevance to include DWI with ADC for response evaluation of treated head and neck tumor patients.

## Introduction

Head and neck tumors are a devastating disease being the seventh leading cancer with respect to incidence, and the eight with respect to mortality rates [1]. Incidence in developing countries compared to developed countries is even higher [2]. Patients with head and neck tumors follow an intensive and expensive treatment regime most often consisting of concomitant chemoradiotherapy. Surgery is not standard in the majority of the patients with locally advanced tumors, but is frequently performed in other patients groups [3]. Side effects of treatment are substantial which impacts quality of life [4]. Furthermore, many of the patients with locally advanced tumors demonstrate an inadequate treatment response [5]. Imaging follow-up is thus essential to evaluate treatment response and to tailor treatment in individual patients.

Conventional anatomical MRI techniques are commonly used for treatment evaluation, but are often not able to reliable identify treatment response [6]. Surgery as well as chemoradiotherapy induces false positive results by changes in the affected area, including fibrosis and necrosis [7]. These benign treatment induced changes should be differentiated from true residual or recurrent tumor on imaging to prevent unjustly discontinuation or initiation of therapy. On the other hand, missing a residual or recurrent tumor also results in inadequate treatment for the patient.

Several recent studies have shown encouraging results using diffusion weighted imaging (DWI) for the detection of recurrent head and neck tumors, including calculated apparent diffusion coefficient (ADC) as potential valuable imaging biomarker for treatment response evaluation [8]. Next, perfusion and magnetic resonance spectroscopy (MRS) are promising techniques [9,10]. This is further supported by a recent overview [11]. However, an overview of the diagnostic accuracy for these advanced MRI techniques is not available as systematic review or meta-analysis [8–11].

This prompted us to conduct a meta-analysis of the diagnostic accuracy of anatomical and advanced MRI techniques for tumor residual or recurrence in patients treated for head and neck tumors. We hypothesized that the advanced MRI techniques perform better than anatomical MRI techniques in the differentiation of tumor from treatment induced imaging changes.

## Methods

Our systematic review was performed according to the Preferred Reporting Items for Systematic reviews and Meta-Analyses (PRISMA, see S1 PRISMA Checklist) criteria and the AMSTAR guidelines [12,13]. Furthermore, the Cochrane handbook for review of diagnostic test accuracy was used. A review protocol was written prior to the study start (available upon request).

### Data sources and search strategy

PubMed, EMBASE and Web of Science were searched by AH and HW in separate sessions using the same search strategy from their first records to September 23^th^ 2014. Database keywords and text words were searched using head and neck tumors, MRI techniques, treatment options and treatment response including the subcategories and variants of these words as search terms (see S1 Text). No filters were used, but studies in non-English languages were excluded manually later. References of included studies were further hand searched. An effort was made to include unpublished data by searching EMBASE for conference proceeding and contacting authors of in case insufficient details were described to generate 2x2 tables.

### Selection criteria

We searched for studies with patients who were treated for newly diagnosed head and neck tumors. Studies reporting on patients with tumors (squamous cell carcinoma) of the oral cavity, pharynx or larynx were included. The reference standards should determine the treatment effect, thus tumor recurrence or e.g. therapy induced changes by clinical follow-up, imaging follow-up, histology or a combination of these. Studies were included if a 2x2 table could be constructed for the anatomical or advanced MRI data using the full text or addition requested data from the authors.

We excluded studies of patients with salivary gland neoplasms, thyroid gland neoplasms, parathyroid neoplasms, facial neoplasms, esophagus neoplasms or tracheal neoplasms. Studies in which a MRI system <1.0 Tesla was used were excluded since data differ substantially from data obtained in the current common clinical practice using MRI systems ≥1.0 Tesla.

### Study selection

Study selection, data extraction and study quality assessment was independently done by two authors (AH and HW) and discrepancies were resolved by discussion. Possible inclusion was assessed first based upon title and secondly based upon abstract. The full text was assessed for eligibility if the abstract suggested relevance. Subsequently, the article was included if it fulfilled the inclusion criteria of our study. References of included studies were hand searched.

### Data extraction and quality assessment

Data extraction was done with the use of a data extraction form. The main data extracted consisted of the number of true positives, false positives, false negatives and true negatives. We further extracted data on study design, total number of patients, number of males/females, mean and range of patients’ age, patient selection criteria, imaging characteristics, reference standard (histology/ imaging follow-up/ clinical follow up) and definition of tumor or treatment changes. In case of incomplete 2x2 tables, the corresponding author was contacted and requested to provide the required data to generate 2x2 tables. The quality of included studies was assessed using the quality assessment of diagnostic accuracy studies, QUADAS-2 [14].

### Data synthesis

Sensitivity and specificity with 95% confidence interval (CI) were generated for anatomical MRI and advanced MRI with RevMan 5.3 (Cochrane collaboration, Copenhagen, Denmark). When several time points were measured in one study, we used the one that was closest to 6 weeks posttreatment for the main analysis, because that is the most commonly used imaging follow-up based on our experience. Furthermore, diagnostic accuracy was evaluated in subgroups for the intratreatment, early posttreatment and late posttreatment scan moment. These were set at 2 weeks after the start of treatment, 6 weeks after the end of treatment and ≥3 months after the end of treatment or the most nearing time.

Bivariate random effects models [15] were used to generate pooled estimates of the sensitivity, specificity, positive likelihood ratio and negative likelihood ratios with a 95% confidence interval for each index test when 5 or more studies were included. The sensitivity and specificity were displayed together with a hierarchical summary receiver operator curve (HSROC). We fitted meta-regression in bivariate models and compared sensitivity and specificity of anatomical MRI versus ADC with a likelihood ratio test. The direct comparisons of MRI techniques per study was tested with a two-sample Z test for proportions. The metandi module was used for meta-analysis of diagnostic test accuracy studies in STATA version 12.1 (College Station, Texas, USA). As suggested by the Cochrane Diagnostic Test Accuracy group, no analyses of study heterogeneity or funnel plot asymmetry were performed, as these tests are inaccurate. However, our used random effects model takes heterogeneity into account. Heterogeneity was assessed by visual inspection of the forest plots. We evaluated whether differences in selection (high risk population versus follow-up of all patients) could explain identified heterogeneity. In case of outliers we evaluated whether bias of specific study characteristics could explain the result and performed sensitivity analysis without the outlier to show the influence on the test outcome.

Potential clinical implication was illustrated by calculating the number of missed tumors and total misclassifications using the pooled sensitivity and specificity results for a hypothetical cohort of 100 patients treated for head and neck tumors. Overall prevalence of tumor residual or recurrence in this cohort was based on the mean prevalence of tumor in our included studies.

## Results

### Description of studies

**Fig 1:**
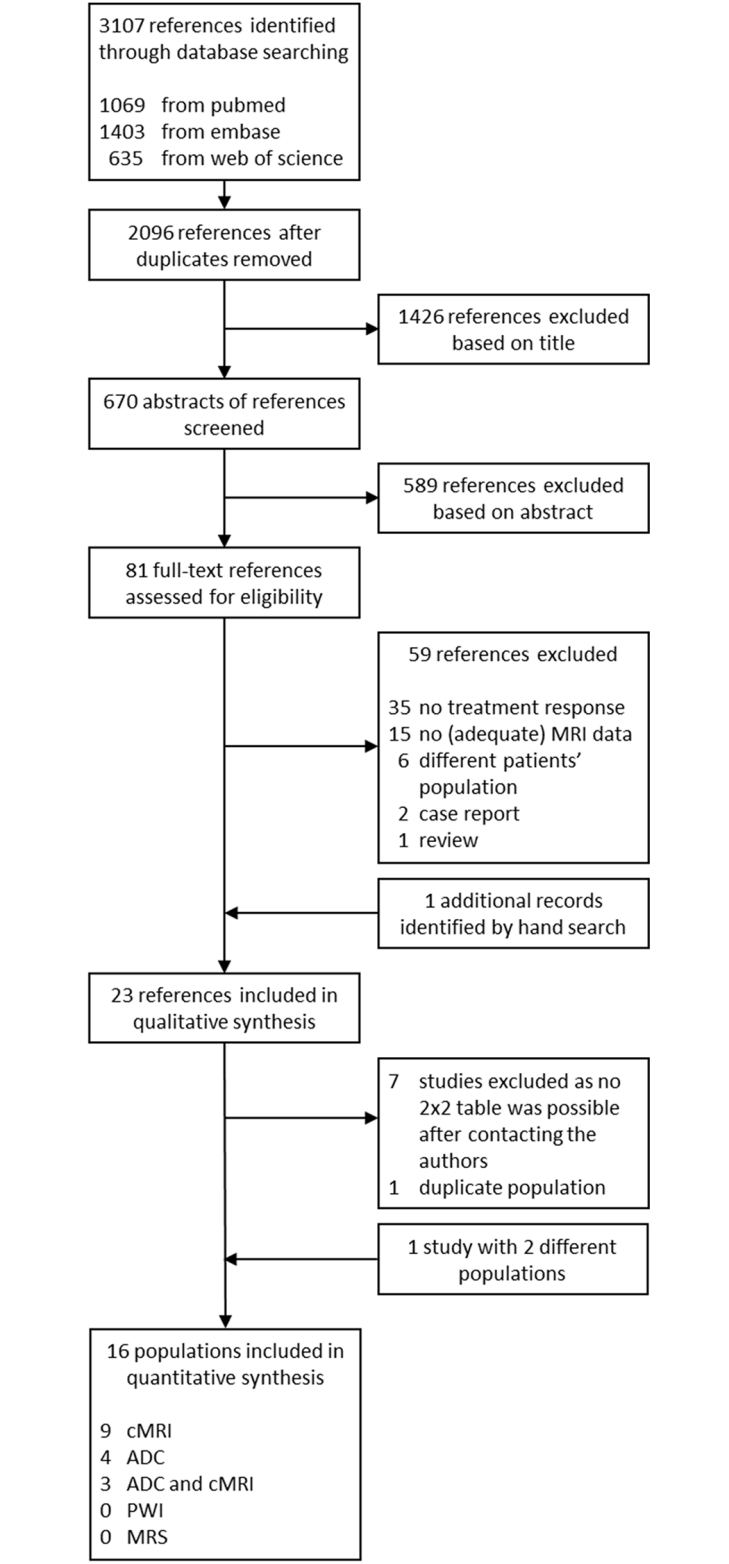
Flow chart literature searches.

Our electronic search revealed a total of 2096 unduplicated references, of which 23 references were eligible for inclusion in the meta-analysis (Fig 1; Tables 1 and 2) [16–38]. Seven references were excluded, because authors were unable to provide the requested information to generate a 2x2 table [32–38]. Two references of the initial 23 were based on the same patient population [29,30] of which data from the first publication was considered to be leading, although both were very similar. One reference described two separate populations of patients [18], group A [18]A and group B [18]B, respectively. This resulted in the inclusion of 16 patients populations (studies) in the meta-analysis with data from 15 references.

**Table 1:** Characteristics of included studies.

Reference	*N*	Study type	Age mean (range)	% male	Tumor locations	Treatment	Selection	Reference standard definition tumor	MRI sequences Field strength; sequence orientation slice thickness/ gap in mm (TR/TE in ms); b values and DWI analysis method	Time point MRI	Diagnostic accuracy	TP	FP	FN	TN
Berrak et al., 2011 (+unpub. data)	18	Retro	57 (48–72)	89	LN of 13 oropharynx; 4 nasopharynx; 1 hypopharynx	18 neoadj chemo + CCRT	All patients with neoadj chemo without other cancers, previous treatment or high comorbidity.	Histology/ imaging or clinical follow-up with responders showing ≥50% reduction and <50% was considered partial responder. All were confirmed with neck dissection at a later stage.	aMRI; 1.5–3.0 T; T1 tra 5/- (600/10); T2 tra 5/- (2000-4000/13, 53, 80, 110, 131); T1C tra 5/-	3 weeks after end neoadj chemo	aMRI LN (T2) aMRI LN (volume) ADC (>50%)	13 1 11	2 0 0	2 14 4	1 3 3
Bhatia et al., 2010	69	Retro	59 (45–75)	91	**Primary site** of 24 oral cavity or oropharynx; 24 hypopharynx; 18 larynx; 3 nasal cavity	69 CCRT	All patient that received CCRT. Patients with <1 year follow-up (*N* = 19) or primary and nodal site not separate (*N* = 1) excluded.	Histology/ definite disease progression on serial MRI	aMRI; 1.5 T; **T1** tra 4/0 (477/12) **T2** fs tra 4/0 (2500/100); **T1C** tra	2 weeks after start treatment; 6 weeks after end treatment	aMRI primary (Δabsolute volume >10.6 cm^3^) aMRI primary (Δabsolute volume >5.7 cm^3^)	13 11	8 4	4 8	23 38
Chan et al., 2006 group A	34	Pros	48 (SD ± 11)	69	**Primary site** of 34 nasopharynx	21 RT; 13 CCRT; 5 addition ICBT	Suspected local recurrence. Exclusion if <6 mo follow-up (*N*≤5) or high glucose (*N*≤1)	Outcome MDT discussion using; histology or if not available >6 mo imaging and clinical follow-up with a 5 point probability scale.	aMRI; 1.5 T; **T1** tra 5/1, sag 4/1 (500/20); **T2** fs tra 5/1, cor 4/1 (3000/85); **T1C** fs tra 5/1, sag 4/1, cor 4/1 (500/20)	17 (6–108) mo after end treatment	aMRI primary	21	3	1	9
Chan et al., 2006 group B	212	Pros	48 (±12)	72	**Primary site** of 112 nasopharynx	19 RT; 93 CCRT; 13 addition ICBT	All nasopharyngeal tumors. Exclusion if <6 mo follow-up (*N*≤5) or high glucose (*N*≤1)	Outcome MDT discussion using; histology or if not available >6 mo imaging and clinical follow-up with a 5 point probability scale.	aMRI; 1.5 T; **T1** tra 5/1, sag 4/1 (500/20); **T2** fs tra 5/1, cor 4/1 (3000/85); **T1C** fs tra 5/1, sag 4/1, cor 4/1 (500/20)	3 mo after end treatment	aMRI primary	3	11	1	197
Chong and Fan, 1997	34	Retro	46 (28–66)	65	**Primary site** of 34 nasopharynx	34 RT	Availability of follow-up, excluded in no follow-up (*N* = 80)	Histology for abnormal clinical or radiology finding; clinical and imaging follow-up for unequivocal clinical or imaging; clinical follow-up for normal clinical and imaging. Recurrence on MRI classified as mass intermediate on T1 with enhancement or high on T2. Borderline imaging were described as mucosal asymmetry.	aMRI; 1.0 T; **T1** tra 5/2 (700/15), cor 5/2 (580/15), sag 4/1 (580/15); **T2** tra 5/2 (2730/80); **T1C** cor 5/2 (580/15), sag 4/1 (580/15)	19 (5–30) mo after end treatment	aMRI primary	5	7	4	29
Comoretto et al., 2008	63	Retro cons	52 (13–79)	70	**Primary site** and **LN** of 63 nasopharynx	63 RT + neoadj chemo	Availability of follow-up	Histology or >6 mo imaging follow-up. Primary site judged by two head and neck radiologists in consensus. LN are metastatic if >10 mm short-axis or >5 mm short-axis for retropharyngeal according to American Joint Committee on Cancer staging criteria for NPC (2002)	aMRI; 1.5 T; **T1** tra 5/0.5 (600/15); **T2** tra 4/0.4 (4200/102); **T1C** fs tra, cor (± sag) 5/0.5 (600/15)	2–14 mo after end treatment	aMRI primary aMRI LN	27 19	4 4	1 2	31 38
Gouhar and El-Harir, 2011	21	Pros	59 (47–66)	76	**Primary site**; 21 larynx	21 RT	Suspected of tumor recurrence without MRI contra-indications.	Histology 2–5 days after MRI	aMRI; 1.5 T; **T1** tra (± cor, sag) 4/0.4 (500-600/8-9); **T2** tra (± cor, sag) 4/0.4 (3000/100); **T1C** tra (± cor, sag) 4/0.4 (500-600/8-9). **DWI**; 1.5T; tra 3-4/1 (2000-2600/64-70); b 0, 1000; ROI	2–6 mo after end treatment	ADC primary 0.85 1.01 1.16 1.49 2.22	— — 11 — —	— — 1 — —	— — 2 — —	— — 7 — —
Hong et al., 2013	134	Pros	47 (18–79)	70	**Primary site** of 134 nasopharynx	121 chemo + IMRT; 13 IMRT	All nasopharyngeal tumors for RT. Excluded if stop or switch of treatment (*N* = 4), not all MRI data acquired (*N* = 13)	Histology or imaging follow-up suggesting residual soft tissues or thickening of the mucous membrane of the nasopharynx with local bulges as indication of residual mass	**DWI**; 1.5 T; (600/min) b 0, 800, ROI	2 weeks after start treatment	ADC primary ΔADC 53%	16	40	7	71
Hwang et al., 2013	33	Retro	60 (30–78)	55	**Primary site** of 16 oral cavity; 4 oropharynx; 5 sinonasal cavity; 3 nasopharynx; 2 hypopharynx; 3 external auditory canal	9 OP; 7 chemo and RT; 13 OP and RT; 4 OP, chemo and RT	Availability of follow-up and new enhancing region suspicious of tumor recurrence or indeterminate and > 6 mm	Histology or imaging follow-up were recurrence was growth of an enhancing lesion (>20% or continuous growth on second follow-up) and posttreatment changes are defined as no further growth in the contrast enhancing area for at least 1 year	aMRI; 1.5 T; **T1** tra 4/1.2 (550-560/10-12); **T1C** fs tra, cor and sag 4/1.2 (550-560/10-12); **DWI**; 1.5 T; tra 4/1.2 (8000-10000/62-78); b 0, 1000, 2000; ROI	> 6 weeks after end treatment, mean 12 mo	ADC primary; ADC 1.46 ADC ratio 63%	17 19	2 4	3 1	11 9
King et al., 2013a	37	Retro	57 (45–71)	92	**Primary site** of 17 oral cavity/ oropharynx; 13 hypopharynx; 5 larynx; 2 esophagus	36 CCRT; 1 RT	Primary tumors collected from two other studies	Histology, endoscopy or serial imaging with increasing mass; no mass (pattern 0), fibrosis with flat-edged/ retracted low signal mass (pattern 1) and indeterminate mass (pattern 3) are compared with focal expansile mass ≥ 1 cm with intermediate T2 signal. Pattern 0, 1 and 2 are negative MRI, pattern 3 is positive MRI.	aMRI; 1.5T; T1; **T2** fs tra 4/0 (2500/100); T1C	6 weeks after end treatment	aMRI primary (T2 pattern)	9	7	0	21
King et al., 2013b	37	Pros	57 (45–71)	86	**Primary site** of 14 oropharynx, oral cavity; 20 hypopharynx, larynx; 2 nasal cavity; 1 maxillary sinus	CCRT or CRT	Biopsy proven untreated stage III or IV tumor. Exclusion if artefacts (*N* = 9), tumor <6 mm (*N* = 2), <2 year follow-up (*N* = 7), or tumor and nodal metastasis not separate (*N* = 1)	Histology or clinical and radiological follow-up with new mass or increasing mass defined as tumor.	aMRI; 1.5 T; T1; T2; T1C **DWI**; 1.5 T; fs tra; 4/0 (2000/75); b 0, 100, 200, 300, 400, 500; ROI	2 weeks after start treatment	ADC primary Skewness (>0.4) Kurtosis (>0.9)	10 10	4 6	3 3	13 11
Ljumanovic et al., 2008	80	Retro	60 (45–71)	79	**Primary site** of 32 supraglottic; 48 glottic	68 RT; 12 neoadj chemo + RT	All larynx SCC patients with RT with curative intent with ≥24 mo follow-up. Otherwise excluded (*N* = 80)	Histology or imaging follow-up with laryngoscopy every 2 mo for the first 2 years. Three point MRI scale with complete resolution of tumor and no asymmetry, focal mass <1 cm or asymmetry or focal mass >1 cm or less than 50% reduction of tumor volume	aMRI; 1.0–1.5 T; **T1** 3-1/1 (310-800/15); **T2** 3-7/1 (2200-4550/90-98); **T1C** 3-7/1 (310-800/15)	5 (1–16) mo after end treatment	aMRI primary	25	13	1	41
Ng et al., 2010	179	Pros	27 (19–84)	89	**Primary site** and **LN** of 179 nasopharynx	174 CCRT; 3 RT; 2 RT + intra-cavity RT	Patients at high risk for recurrence or with suspected recurrence	Histology for suspected lesion if possible or imaging follow-up for at least 12 months. MRI with 5 point probability scale	cMR; 3.0 T; **T1** tra 4/2 (562/10); **T2** fs tra 4/2 (6640/88); **T1C** tra 4/2 (550/10), cor 4/2 (600/10)	6,5 (3–25) mo after end treatment	aMRI primary aMRI LN	25 22	7 5	4 3	143 149
Tshering Vogel et al., 2013	46	Pros	60 (41–83)	89	**Primary site** of hypopharynx 16; larynx 30	16 RT; 7 OP + RT; 19 chemo + RT; 1 OP + chemo + RT; 1 RT + LR; 2 OP + LR + RT + chemo	Patients with new or worsening symptoms after treatment. Excluded if susceptibility artefacts (*N* = 4)	Histology or imaging follow-up of at least 1 year with focal enhancement or increase in size of lesion was considered tumor on aMRI and high DWI with low ADC for diffusion MRI.	aMRI; 1.5 T; **T1** tra 3/0.6 (624/12); **T2** tra 3/0.6 (3630/76); **T1C** fs tra 3/0.6 (624/12, cor and sag 3/0,75 (630/18) **DWI**; 1.5 T; tra 3/0.6 (3500/69); b 0, 50, 100, 500, 750, 1000; ROI	31 (2–96) mo after end treatment	aMRI primary ADC primary, ADC visual ADC_T_ (1,30) ADC_D_ (1,30) F_p_ (23%)	13 17 12 14 17	12 0 3 6 6	5 1 6 4 1	16 21 18 15 15
Vandecaveye et al., 2010 and Vandecaveye et al., 2012	30	Pros	53 (38–66)	93	**Primary site** and **LN** of 5 tonsil; 7 piriform sinus; 7 supraglottic; 3 base of tongue; 6 oropharynx	27 CCRT, 3 RT	All patients with histological proven SCC. Exclusion if distant metastasis before treatment (*N* = 1) or claustrophobia (*N* = 1).	Histology of imaging follow-up for 2 years with volume increase of persisting mass ≥ 65% and recurrent mass indicating tumor. MRI scoring of primary lesion was done on 3 point scale, no focal abnormality, asymmetry or mass <10 mm and mass >10 mm or <50% reduction.	aMRI; 1.5 T; **T1** tra 4/0.4 (775/8.3); **T2** tra 4/0.4 (3080/106); **T1C** tra, cor, sag 4/0.4 (775/8.3) DWI; 1.5 T; tra 4/0.4 (7100/84); b 0, 50, 100, 500, 750, 1000; ROI	2 weeks after start treatment 4 weeks after start treatment 3 weeks after end treatment	aMRI primary Δvolume 20%; ADC primary ΔADC 14%; aMRI LN Δvolume 33%; ADC LN ΔADC 15% aMRI primary Δvolume 65%; ADC primary ΔADC 25%; aMRI LN Δvolume 50%; ADC LN ΔADC 19% aMRI primary ADC primary ΔADC 25%; aMRI LN ADC LN ΔADC 20%	7 7 9 8 6 8 7 8 6 8 6 7	10 2 23 5 10 2 17 2 6 1 11 5	1 1 1 2 2 0 3 2 2 0 3 2	13 21 21 39 13 21 27 42 16 21 30 36
Yen et al., 2003	67	Pros	47 (16–75)	79	**Primary site** of 67 nasopharynx	RT or CCRT	Patients with clinical suspicion of residual or recurrence. Exclusion if pregnant or diabetic.	Histology for positive PET or MRI findings or clinical follow-up >6 mo for others. MRI done by visual interpretation not specified.	aMRI; 1.5 T; **T1** cor, sag; **T2** tra; **PD** tra; **T1C** fs tra, cor; T1C tra	4–70 mo after end treatment	aMRI primary	13	26	8	20

Characteristics of the 15 included studies are shown. Abbreviation: aMRI = anatomical MRI; OP = operation; RT = radiotherapy; CCRT = concomitant chemoradiotherapy; tra = transversal; cor = coronal; sag = sagittal; mo = months; TP = true positive; TN = true negative; FP = false positive; FN = false negative; SCC = squamous cell carcinoma; TR = repetition time; TE = echo time; T = Tesla; ICBT = intracavitary brachytherapy; IMRT = intensity modulated radiotherapy; ca = carcinoma; undif = undifferentiated; neoadj = neoadjuvant; chemo = chemotherapy; ROI = region of interest analysis; min = minimal; LR = laser resection; mm = millimeter; ms = milliseconds; ADC cut-off (x10^-3^ mm^2^/s); pros = prospective; retro = retrospective; LN = lymph nodes; cons = consecutive

**Table 2:** Characteristics of excluded studies.

Reference	*N*	Study type	Age mean (range)	% male	Tumor locations	Treatment	Selection	Reference standard definition tumor	MRI sequences Field strength; sequence orientation slice thickness/ gap in mm (TR/TE in ms); b values and DWI analysis method	Time point MRI	Diagnostic accuracy	TP	FP	FN	TN
Chen et al., 2014	31	Pros	45 (18–68)	84	**Primary site** and **LN** of 31 nasopharynx	29 CCRT; 2 RT	All patients with no prior treatment stage III/IV. Excluded if distant metastases before treatment (*N* = 4) or MRI artefact (*N* = 2)	Imaging follow-up according to RECIST 1.1 and residual disease was classified by residual soft tissues or thickening of the mucous membrane of the nasopharynx with local bulges	aMRI; 3.0 T; **T1** fs tra, sag; **T2** tra 5/1; **T1C** fs tra, cor **DWI**; 3.0 T; 5/1 (6600/70); b 0, 800; ROI	3 days after start treatment; 20 days after start treatment; 50 days after start treatment	ADC primary ADC LN ADC primary ADC LN ADC primary ADC LN	— — — — — —	— — — — — —	— — — — — —	— — — — — —
Galbán et al., 2009	15	Pros	—	—	**Primary site** (12) and **LN** (14) of head and neck	15 CCRT	All patients for CCRT. Exclusion if claustrophobic (*N* = 2), metal implants (*N* = 2), withdrew from study (*N* = 3), inadequate DWI (*N* = 1).	Histology or imaging 2 mo after end treatment and clinical outcome at 6 mo.	aMRI; 3.0 T; **T1**; 1/0 (9.9/4.6); **T2** fs 4/- (5000/120); **T1C**; 1/0 (9.9/4.6) **DWI**; 3.0 T; 4/- (2789/59); b 0, 800; ROI	3 weeks after start treatment	ADC primary ADC LN aMRI primary (Δvolume %) aMRI LN (Δvolume %)	— — — —	— — — —	— — — —	— — — —
Kim et al., 2009	33	Pros	61 (31–78)	79	**LN** of 11 base of tongue; 10 tonsil; 6 larynx; 1 vallecula; 5 unknown	24 CCRT; 7 RT + immune-therapy	All head/neck cancers with preoperative CCRT and with metastatic lymph nodes. Excluded if death unrelated to treatment (*N* = 4), claustrophobia (*N* = 1), withdraw (*N* = 1) or artefact on DWI (*N* = 1)	Histology or 6 mo clinical/ imaging follow-up	aMRI; 1.5–3.0 T; **T1** tra (600/10); **T1C**; tra (300/4); **T2** tra 5/- (4000/120) and (2000/13, 53, 80, 110); **DWI**; 1.5-3T; 5/- (40000/89); b 0, 500, 1000; ROI	1 week after start treatment; 2 weeks after end treatment	ADC LN aMRI LN T2 volume ADC LN aMRI LN T2 volume	— — — — — —	— — — — — —	— — — — — —	— — — — — —
King et al., 2010	50	Pros	58 (45–73)	90	**Primary site** of 9 oral cavity/ oropharynx; 13 hypopharynx; 4 larynx; 1 maxillary sinus; 2 nasal cavity; and 21 **LN** of not described primary	44 CCRT; 6 RT	All biopsy proven tumors with curative intent of CCRT or RT with stage III or IV. Excluded in no consent (*N* = 14), artefacts (*N* = 10) or death before definitive diagnosis (*N* = 4)	Histology for the primary site and histology or increase in size on serial imaging follow-up >12 mo for lymph nodes; fall in ADC as local failure	aMRI; 1.5 T; T1; T2; T1C. **DWI**; 1.5 T; fs tra; 4/0 (2000/75); b 0, 100, 200, 300, 400, 500; ROI	6 weeks after end treatment;	Primary/LN ADC 1.4	6	0	1	13
Lell et al., 2000	39	—	—	—	**Primary site** of 21 oropharynx, oral cavity; 1 naso-pharynx; 17 hypo-pharynx, larynx	CCRT	Locally advanced tumor with CCRT	Histology or clinical follow-up with MRI-based tumor recurrence classified as a localized expansive mass with intermediate signal on T1 and high on T2 with marked enhancement	aMRI; 1.5 T; **T1** tra, cor 6/0.6 (500-600/20-30); **T2** tra 6/0.6 (2500-3500/30-100); **T1C** tra,cor 6/0.6 (500-600/20-30); **IR** cor 6/0.6 (1400/30)	<3 mo after treatment end; 3–6 mo after treatment end	aMRI primary aMRI primary	13 5	— —	— —	11 7
Matoba et al., 2014	35	Pros	66 (33–79)	86	**Primary site** and **LN** of 4 oral cavity; 9 oropharynx; 9 hypopharyx; 10 larynx; 3 supraglottic	35 CCRT	All patients treated with CCRT with curative intent. Excluded if patient refuse treatment (*N* = 2), poor imaging quality (*N* = 2) or death within 3 mo after treatment (*N* = 1)	Imaging follow-up every 6 mo with increasing mass or histology proof as indication of tumor recurrence	aMRI; 1.5 T; **T1** tra (630/12); **T2** tra, cor (4000/90); **DWI**; 1.5 T; fs tra 6/3 (4000/68); b 0, 90, 800	3 weeks after start treatment	ADC primary ΔADC 0.24 ADC ADC LN ΔADC 0.24 ADC aMRI primary Δvolume aMRI LN Δvolume	— — — — — —	— — — — — —	— — — — — —	— — — — — —
Mukundan et al., 2014	50	Pros	56	80	**Primary site** of 50 head and neck	11 OP; 26 RT; 13 OP + RT	All patients with treatment without previous treatment and no significant comorbidity	Histology	aMRI; 1.5 T; **T1** with and without fs 3-4/0-1; **T2** 3-4/0-1; **T1C** fs 3-4/0-1	12 weeks after end treatment; 24 weeks after end treatment	aMRI primary aMRI primary	— —	— —	— —	— —

Characteristics of the 7 excluded studies are shown. See Table 1 for abbreviations.

The included diffusion studies concerned 1087 patients with a mean age of 48 years and of whom 78% was male. Mean tumor prevalence was 25% (range 2–83), without differences for during treatment (range 9–21) or posttreatment tumor prevalence (range 2–83). As the tumor prevalence was overlapping for studies that performed follow-up of all patients (prevalence range 16–65) and studies that selected patients with a suspicion of tumor recurrence or a high risk population (prevalence range 2–83), we combined these groups in the further analysis. As some studies described both anatomical and advanced MRI or both primary and nodal sites, we had a total of 11 studies (854 patients) for anatomical MRI of the primary tumor site, 6 ADC studies of the primary site (287 patients), 4 anatomical MRI studies of the nodal sites (310 patients) and 2 ADC studies of the nodal site (68 patients). No studies concerning perfusion or spectroscopy MRI were available. The definition for differentiating tumor from treatment effects was variable between studies and described for each separately (Tables 1 and 2).

### Methodological quality of included studies

**Fig 2:**
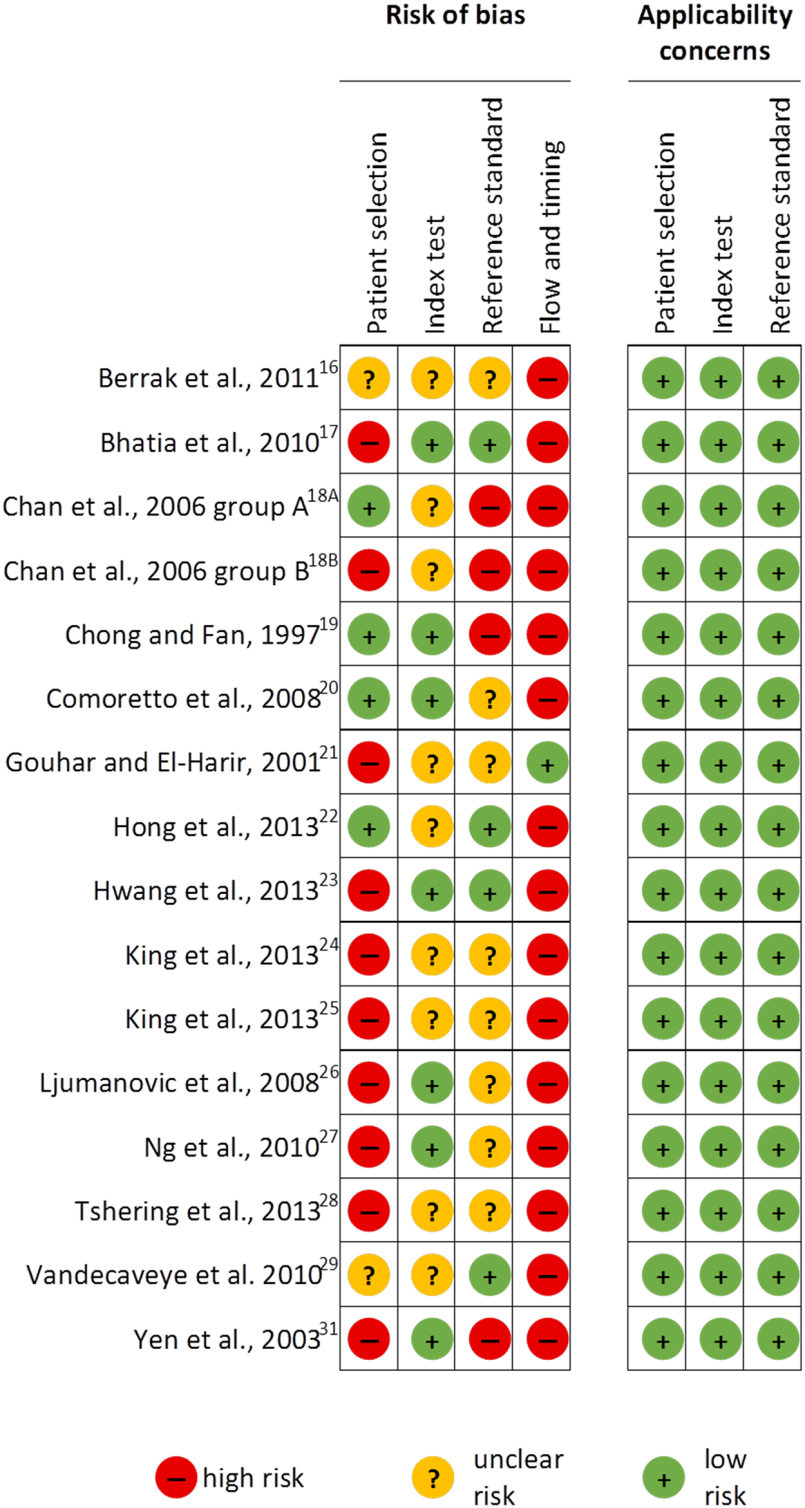
Risk of bias and applicability concerns summary with for each domain of the QUADAS-2 for each included study.

The methodological quality of the included studies is summarized (Fig 2). In the patient selection domain, four studies were considered to be of high risk of bias due to inappropriate exclusion criteria as patients with less than a 1 year disease-free follow-up [17] or patients with less than 2 year follow-up of the primary site were excluded in these studies [24–26]. Requiring such a long disease-free period creates a selection bias favoring patients without tumor recurrence. We considered another six studies to be at high risk of bias because a non-random selection was carried out as patients with at high risk for recurrence or with a suspicion of recurrence were included only [18]B,[21,23,27,28,31]. Moreover, in one study it was only stated that they included nasopharyngeal carcinomas without explicitly mentioning the inclusion of squamous cell carcinomas, providing an additional argument to classify this study as high risk [31]. Two studies were classified as being of unclear risk of bias due to poor reporting of inclusion and exclusion criteria [16,29]. The remaining four studies were considered to be at low risk of bias [18]B,[19,20,22].

In the index test domain a total of nine studies were considered to be of unclear risk as it was not described whether the results were interpreted without the knowledge of the reference standard [16,18]A,[18]B,[19,21,22,24,25,28,29]. The other seven studies were classified as low risk [17,19,20,23,26,27,31].

In the reference standard domain, four studies were classified as being of high risk because the index test results were known when interpreting the reference standard [18]A,[18]B,[19,31]. Eight studies were judged as unclear risk because it was unclear whether the results of the index test were known during the interpretation of the reference standard [16,20,21,24–28]. The remaining four studies were considered to be at low risk of bias [17,22,23,29].

In the flow and timing domain, 15 studies were considered to be of high risk because not all patients received the same reference standard [16–20,22–29,31]. Although, a potential bias might be mild as surgery and imaging follow-up are both likely to provide the correct diagnosis. The one remaining study was classified as low risk [21].

Thus, as most studies showed high risk of bias in the domains patient selection and flow and timing and the index test and reference standard domains were mostly unclear, study quality was classified as low.

For assessment of applicability, the included participants and setting, the conduct and interpretation of the index test, and the reference standard in each of the included studies were not doubted to meet the review question. All studies fulfilled the inclusion criteria of the review.

### Main findings primary site

The forest plot of the anatomical MRI (11 studies with 854 patients) for the primary tumor location showed a reasonable homogenous specificity (see S1 Fig). The sensitivity showed more variation in CI, which were wide in 2 studies [18]B,[19]. No outliers were detected.

**Fig 3:**
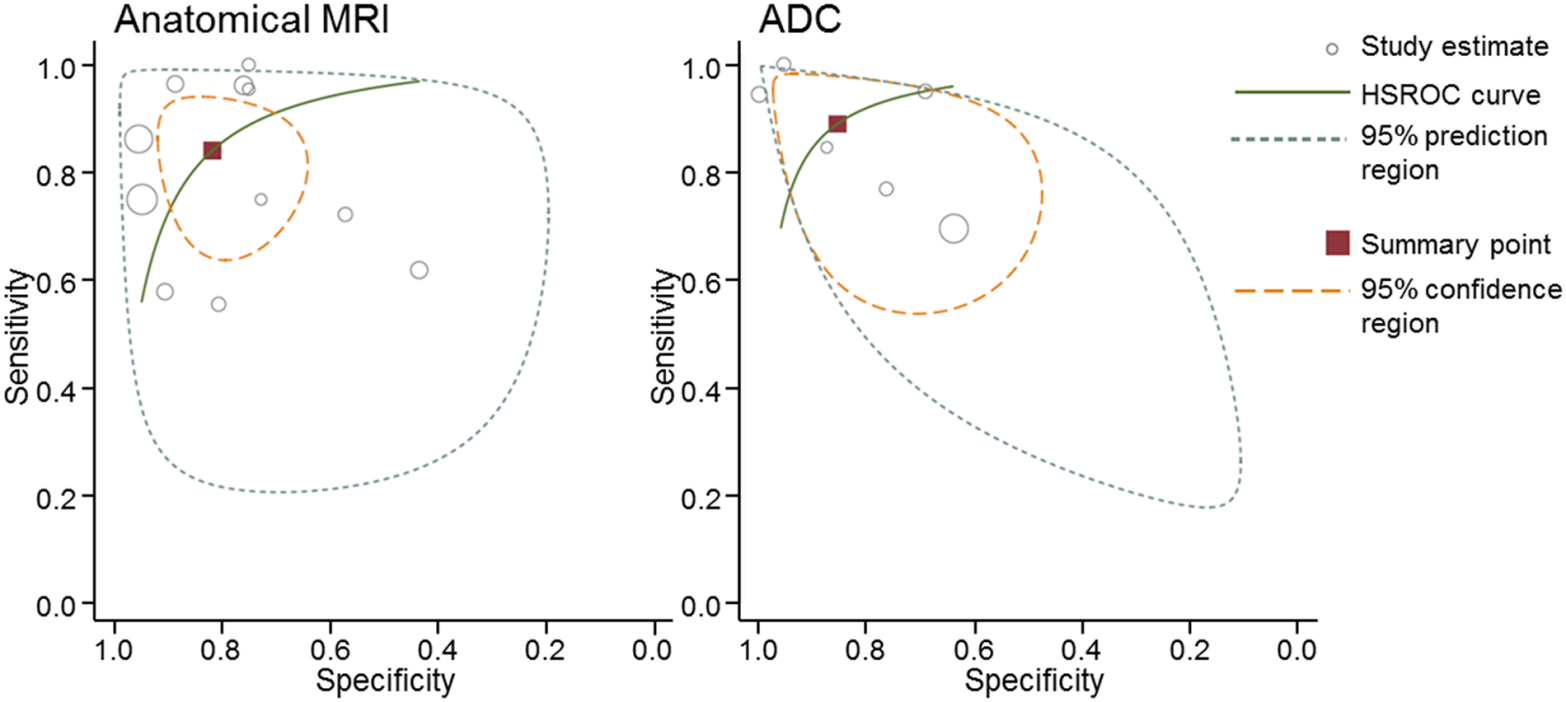
Hierarchical summary receiver operator curves of anatomical MRI and ADC for the primary tumor site.

Pooled results for anatomical MRI and ADC were calculated for the primary tumor location (Table 3 and Fig 3). Pooled anatomical MRI results demonstrated a sensitivity of 84% (95% CI 72–92) and a specificity of 82% (95% CI 71–89). The positive likelihood ratio was 4.6 (95% CI 2.7–7.9) and the negative likelihood ratio was 0.19 (95% CI 0.10–0.37).

**Table 3:** Pooled diagnostic accuracy results.

	Studies	*N*	Preva-lence (%)	Sensitivity (95% CI)	Specificity (95% CI)	Positive LR (95% CI)	Negative LR (95% CI)	Missed tumors progress.	Incorrect treatment	Total misclas-sification
aMRI primary	11	854	23	84 (72–92)	82 (73–89)	4.6 (2.7–7.9)	0.19 (0.10–0.37)	4	13	17
ADC primary	6	287	27	89 (74–96)	86 (69–94)	6.1 (2.5–15.1)	0.13 (0.05–0.34)	3	10	13

Pooled diagnostic accuracy results are shown for the anatomical MRI (aMRI) and apparent diffusion coefficient (ADC) for the primary location of the head and neck tumors. Abbreviations: CI = confidence interval; LR = likelihood ratio; *N* = number.

The forest plot from the ADC (6 studies with 287 patients) showed overlapping confidence intervals for both the sensitivity and specificity. A higher pooled diagnostic accuracy was shown (Table 3 and Fig 3) with a sensitivity of 89% (95% CI 74–96), specificity of 86% (95% CI 69–94), positive likelihood ratio of 6.1 (95% CI 2.5–15.1) and negative likelihood ratio of 0.13 (95% CI 0.05–0.34).

Although the pooled sensitivity and specificity of ADC were higher, this difference was not significant (*p* = 0.457 and *p* = 0.626, respectively). Two studies compared the anatomical MRI for the primary site with the ADC directly. The first study demonstrated a sensitivity of 72% for anatomical MRI and a sensitivity of 94% for ADC (*p* = 0.079). Specificity of both tests were 57% and 100%, respectively (*p* = 0.002) [28]. The second study showed a sensitivity of 75% for anatomical MRI and a sensitivity of 100% for ADC (*p*<0.023) and a specificity of 73% and 95%, respectively (*p*<0.047) [29].

To illustrate the clinical implication of our findings, we calculated the number of missed tumors and the number of total misclassified patients in a hypothetic population of 100 head and neck patients with using the residual or recurrent tumor prevalence of 25% found in our meta-analysis. This calculation showed that follow-up with anatomical MRI would result in 4 missed tumors and 13 patients would receive unjustified treatment. Implementation of ADC maps, however, would reduce the number of missed tumors to 3 and the number of patients that receive unjustified treatment to 10.

### Main findings nodal site

The forest plot of the data for the nodal site for anatomical MRI (4 studies with 310 patients) showed small overlapping confidence interval for the sensitivity and specificity with exception of the sensitivity of one study [29] and the specificity of another study [16] (see S1 Fig). Nodal sites of the anatomical MRI showed a sensitivity range of 67–90% and a specificity range of 33–97%, but there were too few studies to calculate pooled estimates. The forest plot of the ADC of the nodal site (2 studies with 68 patients) showed overlapping, but wide confidence intervals. ADC showed a sensitivity range of 73–78% and a specificity range of 88–100%.

Two studies compared the nodal site directly [16,29]. The sensitivity was 67% and the specificity was 73% for anatomical MRI, for the first study [29]. The ADC demonstrated a non-significant higher diagnostic accuracy with a sensitivity and specificity of 78% and 88%, respectively (*p* = 0.601 and *p* = 0.087, respectively) [29]. Similar results were demonstrated by the second study with a sensitivity of 87% with a specificity of 33% for the anatomical MRI and 73% and 100% for ADC, respectively (*p* = 0.338 and *p* = 0.082) [16].

### Imaging time point

Intratreatment evaluation (2 studies with 79 patients), early posttreatment evaluation (3 studies with 128 patients), and late posttreatment evaluation (8 studies with 726 patients) measurements demonstrated similar diagnostic accuracy for the primary tumor locations for the anatomical MRI (S2 Fig). The sensitivity range was 76–88%, 58–100% and 56–96%, respectively. The specificity range was 57–74%, 73–90% and 43–95%, respectively. This was further supported by the pooled results for the late posttreatment point with a sensitivity of 86% (95% CI 73–93), specificity of 82% (95% CI 67–91), positive likelihood ratio of 4.8 (95% CI 2.3–9.7) and negative likelihood ratio of 0.17 (95% CI 0.08–0.37).

Intratreatment (3 studies with 218 patients), early posttreatment (1 study with 30 patients) and late posttreatment (3 studies with 93 patients) diagnostic accuracy values were also comparable for the ADC studies (S2 Fig) but on average higher than for anatomical MRI. The sensitivity was 70–80%, 100% and 85–95%, respectively. The specificity was 64–89%, 95% and 69–100% respectively.

## Discussion

By using the statistical strategy of a systematic meta-analysis, we were able to demonstrate a benefit of DWI with derived ADC data over anatomical conventional MRI sequences. Pooled ADC values showed a higher sensitivity (89%) and specificity (86%) than anatomical MRI for the primary site (84% and 82%, respectively), while similar results were demonstrated for the fewer studies concerning nodal sites. The higher sensitivity and specificity of ADC values for tumor recurrence is also confirmed by the few available direct comparisons.

The relation between the performance of the anatomical MRI and ADC has been unclear till now as most studies reported diagnostic accuracy data of only anatomical MRI [17–19, 24,26,27,31] or of only ADC data [16,21–23]. Only few have investigated both, but in only 2 studies the diagnostic accuracy was reported of both the anatomical MRI and DWI with derived ADC data for the primary tumor site [28,29]. These direct comparisons are less prone to bias than indirect comparisons. Both studies confirmed the higher diagnostic accuracy of ADC data over anatomical MRI found in our meta-analysis for the primary site with a statistically significant higher sensitivity and specificity for the ADC [28,29]. A similar higher diagnostic accuracy was displayed in the two studies with a direct comparison for the nodal site, although not statistically significant [16,29].

Different ADC thresholds for the differentiation between treatment effects and tumor residual/recurrence were used ranging from 1.16–1.46 x10-3 mm^2^/s for absolute values or 14–53% for relative differences (see also Table 1). This implies that used thresholds cannot be interpolated across hospital sites. Even within studies different cut-off values were used [29,30]. ADC values are also known to show intratumoral variation with low ADC values for solid tumor components and high ADC values for necrotic areas, which can be a caveat in drawing regions of interest [39]. This might be the reason for the different strategies used in the region of interest analyses. Whole tumor volume possibly included necrotic areas [22]. The studies targeting the most conspicuous area can be assumed to exclude necrosis [23], while necrosis is certainly excluded for the studies stated to target the most conspicuous area excluding necrosis [27] or the complete solid component excluding necrosis [16,29]. One study did not provided details about the region of interest analysis, hindering a judgment about the quality [21].

Despite the variation in thresholds, tumor heterogeneity and different b-values, ADC data still outperformed anatomical MRI techniques. Because of the limited number of studies we were not able to assess the diagnostic accuracy of ADC and MRI in various threshold subgroups. However, implementation in clinical practice would benefit from standardized and validated ADC threshold values and region of interest analysis. This lack of standardization and the current high variability also hinders the generation of an advice regarding the best cut-off value to be used in clinical practice. Nevertheless, this meta-analysis demonstrate what many radiologist experience in daily practice, namely that adding a diffusion sequence to the anatomical sequences enhances treatment evaluation.

Numbers of excluded patients due to susceptibility artefacts in the head and neck area were provided in some studies (see Tables 1 and 2). This is a known limitation of DWI sequences, but the current limited data suggest that it is a problem in a minority of the patients. Small primary tumor size was an exclusion criteria in only two studies [23,25]. The sensitivity and specificity reported in studies excluding tumors smaller than 6 mm, however, did not show a significantly higher accuracy over studies without size limitations. Other factors, like claustrophobia played a minimal role.

Data for perfusion and spectroscopy studies were searched, but were not available yet for inclusion in our meta-analysis. Perfusion is, however, feasible and already shows to be able to predict survival before treatment or predict tumor response early in the treatment [9,40]. The potential value of perfusion is also shown by high diagnostic accuracies in treatment response evaluation in patients with brain tumors [41]. Spectroscopy is even less studied although its feasibility has been demonstrated in head and neck tumors. However, diagnostic accuracy remains speculative currently [10].

The main analysis included predominantly posttreatment studies, but also a few intratreatment studies. Combining both was considered to be justified as MRI aims in both to identify viable tumor, although the question differs slightly. Intratreatment MRI aims at differentiating responders from non-responders to adapt the treatment in non-responders, while posttreatment MRI is used to select patients for addition therapy when tumor is shown. The overlapping diagnostic accuracy supports the legitimacy of combining intratreatment and posttreatment MRI.

Identifying non-responders and responders early after treatment start or even before treatment would be optimal. The few intratreatment studies in our data suggest a preference for using ADC data over anatomical MRI for this [17,22,29]. Predicting treatment response before the start of it also favors ADC for primary and nodal sites [34,37,42]. Although good, the performance is until now too variable for wide clinical implication. It might probably benefit from more precise coregistration to anatomical MRI, but also more clinical trials in a large population for validation of DWI early after the start of treatment [43]. Identifying the non-responders with ADC as a potential biomarker early during treatment may enable treatment tailoring and may avoid possible side-effects of an ineffective and expensive treatment regime [44]. Prediction of clinical outcome would be of interest as well.

FDG-PET is frequently used for treatment response assessment with high sensitivity but lower specificity [45]. Compared to FDG-PET, ADC can be performed earlier to assess treatment response. FDG-PET is less reliable in the first months after treatment with false positive results due to inflammation, granulation and scar tissue [46]. ADC can be performed in this period, but false positive and false negatives are not fully excluded. True restricted diffusion can be seen in an abscess or with inflammation, although central enhancement as shown in tumor would be lacking. Scar tissue can display low ADC but normally in combination with lack of diffusion restriction. This distinguishes scar tissue from tumor with low values on the ADC map together with diffusion restriction [47]. Minimal to absent enhancement of scar tissue helps in further differentiation from tumor. Included studies used ADC values only for calculations and therefore likely underestimated the accuracy of diffusion weighted MRI. Combining anatomical MRI with diffusion weighted MRI including *b*-maps, ADC maps and post contrast images would probably demonstrate even higher diagnostic accuracy in clinical practice. The higher specificity (less false positives) of ADC compared to anatomic MRI results in a reduction of unnecessary and costly initiation of treatment in patients with treatment related changes. It might also reduce the patients that are false interpreted on anatomical MRI as having tumor progression resulting in incorrect continuation of therapy. Moreover, the higher sensitivity (less false negatives) of ADC contributes in decreasing the number of missed patients with tumor recurrence.

Multimodal imaging with PET/MR systems is a potential area for further research to increase diagnostic accuracy of treatment response both early after start treatment as well as later posttreatment [47].

In general, the methodological quality of the included studies was similar, but low. This might also explain the wider confidence interval in some studies [18]B, but could not provide a convincing explanation for others [16,19,29]. The heterogeneity of patient selection, reference standards or relatively small group size might provide additional sources of variation. This is a reflection of the complexity of the field, however this variation is an important limitation of the current study. Especially the variability in the definition used to identify tumor residual or recurrence compared to treatment effects as shown in Tables 1 and 2 might be a limiting factor. Furthermore, as discussed above and also displayed in these tables, different b-values and ADC thresholds were used in the different studies. Although it still can be concluded that ADC helps in the differentiation of tumor residual or recurrence and treatment related effects as fibrosis, this variability hinders stronger conclusions and a firm implication in clinical practice. Further research should also focus on comparing all imaging techniques in the same population using direct comparisons to ensure a higher quality. In such a study, the same reference standard should be applied in a consecutive large cohort of patients. This would also allow subgroup analyses to search for the sources of heterogeneity in the diagnostic performance of the MRI sequences.

## Conclusions

To conclude, a higher diagnostic accuracy of ADC values over anatomical MRI in patients with treated head and neck tumors is demonstrated in this meta-analysis. It is should be kept in mind that this was only statistically significant for the direct comparison of the primary tumor site and not convincing for the direct comparisons of the nodal site. However, this emphases the relevance to include DWI with ADC for response evaluation of treated head and neck tumor patients.

## Supporting information

S1 Text Search strategy. (DOCX)

S1 PRISMA Checklist (DOC)

S1 Fig Forest plots with diagnostic accuracy anatomical MRI and ADC for different scan times for the primary tumor site. Diagnostic accuracy and the 2x2 table is displayed with true positives (TP), false positives (FP), false negatives (FN) and true negative (TN). Sensitivity and specificity with the 95% Confidence intervals (CI) are given.
(PDF)

S2 Fig Forest plots with diagnostic accuracy anatomical MRI and ADC for different scan times for the primary tumor site. See caption S1.
(PDF)

## References

[1] Ferlay J, Shin HR, Bray F, Forman D, Mathers C, Parkin DM. Estimates of worldwide burden of cancer in 2008: GLOBOCAN 2008. *Int J Cancer* 2010;127(12):2893–2917. 10.1002/ijc.25516 21351269

[2] Bray F, Jemal A, Grey N, Forman D. Global cancer transitions according to the Human Development Index (2008–2030): a population-based study. *Lancet Oncol* 2012;13(8):790–801. 10.1016/S1470-2045(12)70211-5 22658655

[3] Bar-Ad V, Palmer J, Yang H, Cognetti D, Curry J, Lunginbuhl A, Tuluc M, et al Current management of locally advanced head and neck cancer: the combination of chemotherapy with locoregional treatments. *Semin Oncol* 2014;41(6):798–806. 10.1053/j.seminoncol.2014.09.018 25499638

[4] Ratko TA, Douglas GW, de Souza JA, Belinson SE, Aronson N. AHRQ Comparative Effectiveness Reviews. Radiotherapy Treatments for Head and Neck Cancer Update [Internet]. Rockville: Agency for Healthcare Research and Quality (US) 15-EHC001-EF 2014. 25590120

[5] Pignon JP, le Maître A, Maillard E, Bourhis J; MACH-NC Collaborative Group. Meta-analysis of chemotherapy in head and neck cancer (MACH-NC): an update on 93 randomised trials and 17,346 patients. *Radiother Oncol* 2009;92(1):4–14. 10.1016/j.radonc.2009.04.014 19446902

[6] Rumboldt Z, Gordon L, Bonsall R, Ackermann S. Imaging in head and neck cancer. *Curr Treat Options Oncol* 2006;7(1):23–34. 16343366 10.1007/s11864-006-0029-2

[7] Al-Shwaiheen FA, Wang SJ, Uzelac A, Yom SS, Ryan WR. The advantages and drawbacks of routine magnetic resonance imaging for long-term posttreatment locoregional surveillance of oral cavity squamous cell carcinoma. *Am J Otolaryngol* 2015;36:415–423. 10.1016/j.amjoto.2015.01.024 25697087

[8] Maroldi R, Ravanelli M, Farina D. Magnetic resonance for laryngeal cancer. *Curr Opin Otolaryngol Head Neck Surg* 2014;22:131–139. 10.1097/MOO.0000000000000036 24614061

[9] Zheng D, Chen Y, Liu X, Chen Y, Xu L, Ren W, et al Early response to chemoradiotherapy for nasopharyngeal carcinoma treatment: Value of dynamic contrast-enhanced 3.0 T MRI. *J Magn Reson Imaging* 2015;41:1528–1540. 10.1002/jmri.24723 25136770

[10] Devpura S, Barton KN, Brown SL, Palyvoda O, Kalkanis S, Naik VM, et al Vision 20/20: the role of Raman spectroscopy in early stage cancer detection and feasibility for application in radiation therapy response assessment. *Med Phys* 2014;41(6):050901. 24784365 10.1118/1.4870981

[11] Bhatnagar P, Subesinghe M, Patel C, Prestwich R, Scarsbrook AF. Functional imaging for radiation treatment planning, response assessment, and adaptive therapy in head and neck cancer. *Radiographics* 2013;33(7):1909–1929. 10.1148/rg.337125163 24224586

[12] Moher D, Liberati A, Tetzlaff J, Altman DG, The PRIAMS group. Preferred reporting items for systematic reviews and meta-analyses: the PRISMA statement. *J Clin Epidemiol* 2009;62(10):1006–1012. 10.1016/j.jclinepi.2009.06.005 19631508

[13] Shea BJ, Hamel C, Wells GA, Bouter LM, Kristjansson E, Grimshaw J, et al AMSTAR is a reliable and valid measurement tool to assess the methodological quality of systematic reviews. *J Clin Epidemiol* 2009;62(10):1013–1020. 10.1016/j.jclinepi.2008.10.009 19230606

[14] Whiting PF, Rutjes AWS, Westwood ME, Mallett S, Deeks JJ, Reltsma JB, et al QUADAS-2: a revised tool for the quality assessment of diagnostic accuracy studies. *Ann Intern Med* 2011;155(8):529–536. 10.7326/0003-4819-155-8-201110180-00009 22007046

[15] Reitsma JB, Glas AS, Rutjes AWS, Scholten RJPM, Bossuyt PM, Zwinderman AH. Bivariate analysis of sensitivity and specificity produces informative summary measures in diagnostic reviews. *J Clin Epidemiol* 2005;58(10):982–990. 10.1016/j.jclinepi.2005.02.022 16168343

[16] Berrak S, Chawla S, Kim S, Quon H, Sherman E, Loevner LA, et al Diffusion weighted imaging in predicting progression free survival in patients with squamous cell carcinomas of the head and neck treated with induction chemotherapy. *Acad Radiol* 2011;18(10):1225–1232. 10.1016/j.acra.2011.06.009 21835649 PMC3168957

[17] Bhatia KSS, King AD, Yu KH, Vlantis AC, Tse GMK, Mo FKF, et al Does primary tumor volumetry performed early in the course of definitive concomitant chemoradiotherapy for head and neck squamous cell carcinoma improve prediction of primary site outcome? *Br J Radiol* 2010;83(995):964–970. 10.1259/bjr/27631720 20965907 PMC3473721

[18] Chan SC, Ng SH, Chang JTC, Lin CY, Chen YC, Chang YC, et al Advantages and pitfalls of 18F-fluoro-2-deoxy-D-glucose positron emission tomography in detecting locally residual or recurrent nasopharyngeal carcinoma: comparison with magnetic resonance imaging. *Eur J Nucl Med Mol Imaging* 2006;33(9):1032–1040. 10.1007/s00259-005-0054-6 16622711

[19] Chong VFH, Fan YF. Detection of recurrent nasopharyngeal carcinoma: MR imaging versus CT. *Radiology* 1997;202(2):463–470. 10.1148/radiology.202.2.9015075 9015075

[20] Comoretto M, Balestreri L, Borsatti E, Cimitan M, Franchin G, Lise M. Detection and restaging of residual and/or recurrent nasopharyngeal carcinoma after chemotherapy and radiation therapy: Comparison of MR imaging and FDG PET/CT. *Radiology* 2006;249(1):203–211. 10.1148/radiol.2491071753 18710963

[21] Gouhar GK, El-Hariri MA. *The Eqyptian Journal of Radiology and Nuclear Medicine* 2011;42:169–175.

[22] Hong J, Yao Y, Zhang Y, Tang T, Zhang H, Bao D, et al Value of magnetic resonance diffusion-weighted imaging for the prediction of radiosensitivity in nasopharyngeal carcinoma. *Otolaryngol Head Neck Surg* 2013;149(5):707–713. 10.1177/0194599813496537 23884282

[23] Hwang I, Choi SH, Kim YJ, Le aL, Yun TJ, Kim JH, et al Differentiation of recurrent tumor and posttreatment changes in head and neck squamous cell carcinoma: application of high b-value diffusion-weighted imaging. *AJNR Am J Neuroradiol* 2013;34(12):2343–2348. 10.3174/ajnr.A3603 23811978 PMC7965197

[24] King AD, Keung CK, Mo FKF, Bhatia KS, Yeung DKW, Tse GMK, et al T2-weighted MR imaging early after chemoradiotherapy to evaluate treatment response in head and neck squamous cell carcinoma. *AJNR Am J Neuroradiol* 2013;34(6):1237–1241. 10.3174/ajnr.A3378 23306012 PMC7964595

[25] King AD, Chow KK, Yu KH, Mo FKF, Yeung DKW, Yuan J, et al Head and neck squamous cell carcinoma: diagnostic performance of diffusion-weighted MR imaging for the prediction of treatment response. *Radiology* 2013;266(2):531–538. 10.1148/radiol.12120167 23151830

[26] Ljumanovic R, Langendijk JA, Hoekstra OS, Knol DL, Leemans CR, Castelijns JA. Pre- and post-radiotherapy MRI results as a predictive model for response in laryngeal carcinoma. *Eur Radiol* 2008;18(10):2231–2240. 10.1007/s00330-008-0986-x 18491097

[27] Ng SH, Chan SC, Yen TC, Lia CT, Chang JTC, Ko SF, et al Comprehensive imaging of residual/recurrent nasopharyngeal carcinoma using whole-body MRI at 3 T compared with FDG-PET-CT. *Eur Radiol* 2010;20(9):2229–2240. 10.1007/s00330-010-1784-9 20393714

[28] Tshering Vogel DW, Zbaeren P, Geretschlaeger A, Vermathen P, de Keyzer F, Thoeny HC. Diffusion-weighted MR imaging including bi-exponential fitting for the detection of recurrent or residual tumor after (chemo)radiotherapy for laryngeal and hypopharyngeal cancers. *Eur Radiol* 2013;23(2):562–569. 10.1007/s00330-012-2596-x 22865270

[29] Vandecaveye V, Dirix P, de Keyzer F, op de Beeck K, vander Poorten V, Roebben I, et al Predictive value of diffusion-weighted magnetic resonance imaging during chemoradiotherapy for head and neck squamous cell carcinoma. *Eur Radiol* 2010;20(7):1703–1714. 10.1007/s00330-010-1734-6 20179939

[30] Vandecaveye V, Dirix P, de Keyzer F, op de Beeck K, vander Poorten V, Hauben E, et al Diffusion-weighted magnetic resonance imaging early after chemoradiotherapy to monitor treatment response in head-and-neck squamous cell carcinoma. *Int J Radiat Oncol Biol Phys* 2012;82(3):1098–1107. 10.1016/j.ijrobp.2011.02.044 21514067

[31] Yen RF, Hung RL, Pan MH, Wang YH, Huang KM, Lui LT, et al 18-fluoro-2-deoxyglucose positron emission tomography in detecting residual/recurrent nasopharyngeal carcinomas and comparison with magnetic resonance imaging. *Cancer* 2003;98(2):283–287. 10.1002/cncr.11519 12872346

[32] Chen Y, Lia X, Zheng D, Xu L, Hong L, Xu Y, et al Diffusion-weighted magnetic resonance imaging for early response assessment of chemoradiotherapy in patients with nasopharyngeal carcinoma. *Magn Reson Imaging* 2014;32(6):630–637. 10.1016/j.mri.2014.02.009 24703576

[33] Galbán CJ, Mukherji SK, Chenevert TL, Meyer CR, Tsien C, Lawrence TS, et al A feasibility study of parametric response map analysis of diffusion-weighted magnetic resonance imaging scans of head and neck cancer patients for providing early detection of therapeutic efficacy. *Transl Oncol* 2009;2(3):184–190. 19701503 10.1593/tlo.09175 PMC2730136

[34] Kim S, Louvner L, Quon H, Sherman E, Weinstein G, Kilger A, et al Diffusion-weighted magnetic resonance imaging for predicting and detecting early response to chemoradiation therapy of squamous cell carcinomas of the head and neck. *Clin Cancer Res* 2009;15(3):986–994. 10.1158/1078-0432.CCR-08-1287 19188170 PMC2673914

[35] King AD, Mo FKF, Yu KH, Yeung DKW, Zhou H, Bhatia KS, et al Squanous cell carcinoma of the head and neck: diffusion-weighted MR imaging for prediction and monitoring of treatment response. *Eur Radiol* 2010;20(9):2213–2220. 10.1007/s00330-010-1769-8 20309553

[36] Lell M, Baum U, Greess H, Nömayr A, Nkenke E, Koester M, et al Head and neck tumors: imaging recurrent tumor and post-therapeutic changes with CT and MRI. *Eur J Radiol* 2000;33(3):239–247. 10699740 10.1016/s0720-048x(99)00120-5

[37] Matoba M, Tuji H, Shimode Y, Toyoda I, Kunginuk Y, Miwa K, et al Fractional change in apparent diffusion coefficient as an imaging biomarker for predicting treatment response in head and neck cancer treated with chemoradiotherapy. *AJNR Am J Neuroradiol* 2014;35(2):379–385. 10.3174/ajnr.A3706 24029391 PMC7965773

[38] Mukundan H, Sarin A, Gill BS, Neelakantan A. MRI and PET-CT: Comparison in posttreatment evaluation of head and neck squamous cell carcinomas. *Med J Armed Forces India* 2014;70(2):111–115. 24843197 10.1016/j.mjafi.2013.12.005 PMC4017189

[39] Chawla S, Kim S, Wang S, Poptani H. Diffusion-weighted imaging in head and neck cancers. *Future Oncol* 2009;5(7):959–975. 10.2217/fon.09.77 19792966 PMC2791671

[40] Baer AH, Hoff BA, Srinivasan A, Galbán CJ, Mukherji SK. Feasibility analysis of the parametric response map as an early predictor of treatment efficacy in head and neck cancer. *AJNR Am J Neuroradiol* 2015;36(4):757–762. 10.3174/ajnr.A4296 25792532 PMC5217856

[41] Galbán CJ, Chenevert TL, Meyer CR, Tsien C, Lawrence TS, Hamstra DA, et al The parametric response map is an imaging biomarker for early cancer treatment outcome. *Nat Med* 2009;15(5):572–526. 10.1038/nm.1919 19377487 PMC3307223

[42] Hatakenaka M, Nakamura K, Yabuuchi H, Shioyama Y, Matsuo Y Ohnishi K, et al Pretreatment apparent diffusion coefficient of the primary lesion correlates with local failure in head-and-neck cancer treated with chemoradiotherapy or radiotherapy. *Int J Radiat Oncol Biol Phys* 2011;81(2):339–345. 10.1016/j.ijrobp.2010.05.051 20832179

[43] Lambrecht M, van Herck H, de Keyzer F, Vandecavey V, Slagmolen P, Suetens P, et al Redefining the target early during treatment. Can we visualize regional differences within the target volume using sequential diffusion weighted MRI? *Radiother Oncol* 2014;110(2):329–334. 10.1016/j.radonc.2013.09.023 24231234

[44] Schindler A, Denaro N, Russi EG, Pizzorni N, Bossi P, Merlotti A, et al Dysphagia in head and neck cancer patients treated with radiotherapy and systemic therapies: Literature review and consensus. *Crit Rev Oncol Hematol* 2015;96(2):372–384. 10.1016/j.critrevonc.2015.06.005 26141260

[45] Sheikhbahaei S, Taghipour M, Ahmad R, Fakhry C, Kiess AP, Chung CH, et al Diagnostic accuracy of follow-up FDG PET or PET/CT in patients with head and neck cancer after definitive treatment: A systematic review and meta-analysis. *AJR Am J Roentgenol* 2015;205(3):629–639. 10.2214/AJR.14.14166 26295652

[46] Varoquaux A, Rager O, Dulguerov P, Burkhardt K, Ailianou A, Becker M. Diffusion-weighted and PET/MR imaging after radiation therapy for malignant head and neck tumors. *RadioGraphics* 2015;35(5):1502–1527. 10.1148/rg.2015140029 26252192

[47] Hustinx R, Lucignani G. PET/CT in head and neck cancer: an update. *Eur J Nucl Med Mol* Imaging 2010;37(3):645–651. 10.1007/s00259-009-1365-9 20187296

