# Supplementary material for: Diagnostic accuracy of magnetic resonance imaging techniques for treatment response evaluation in patients with head and neck tumors, a systematic review and meta-analysis
Source: PLoS One. 2017 May 24;12(5):e0177986. doi: 10.1371/journal.pone.0177986 (PMC5443521; doi:10.1371/journal.pone.0177986)
Supplement: S1 Text — (DOCX) [file pone.0177986.s001.docx]

**APPENDIX (online only material)**

**Search strategy pubmed:**

("Head and Neck Neoplasms"[Mesh:NoExp] OR "Mouth Neoplasms"[Mesh] OR "Otorhinolaryngologic Neoplasms"[Mesh] OR ((head and neck[tw] OR pharyn*[tw] OR nasopharyn*[tw] OR oropharyn*[tw] OR hypopharyn*[tw] OR oral cavity[tw] OR laryn*[tw] OR glott*[tw] OR supraglott*[tw] OR subglott*[tw] OR tongue[tw] OR pallat*[tw]) AND (neoplasm*[tw] OR cancer[tw] OR tumor*[tw] OR tumour*[tw] OR carcinoma*[tw])))

**AND** (“magnetic resonance imaging”[MesH] OR MRI [tw] OR “magnetic resonance imaging”[tw] OR diffusion[tw] OR DWI [tw] OR perfusion[tw] OR dynamic contrast[tw] OR contrast enhanced[tw] OR "dynamic susceptibility contrast"[tw] OR DSC[tw])

**AND** (differentiat*[tw] OR differential[tw] OR discriminat*[tw] OR treatment-induced[tw] OR posttreatment[tw] OR post treatment[tw] OR posttherapeutic[tw] OR post therapeutic[tw] OR post irradiation[tw] OR post radiation[tw] OR postradiation[tw] OR residual[tw] OR necrosis[tw] OR necrotic[tw] OR “radiation injuries”[tw])

**AND** (recurren*[tw] OR true[tw] OR response[tw]OR treatment outcome [tw] OR disease progression[tw])

**Search strategy EMBASE:**

((‘head and neck tumor’/de OR ‘head and neck cancer’/de OR ‘head and neck carcinoma’/de OR ‘head and neck squamous cell carcinoma’/de OR ‘head cancer’/de OR ‘mouth cancer’/de OR ‘neck cancer’/de OR ‘tongue cancer’/de OR ‘tonsil cancer’/de OR ‘larynx tumor’/de OR ‘larynx cancer’/exp OR ‘pharynx cancer’/exp) OR ((‘head and neck’:ab,ti OR pharyn*:ab,ti OR nasopharyn*:ab,ti OR oropharyn*:ab,ti OR hypopharyn*:ab,ti OR ‘oral cavity’:ab,ti OR laryn*:ab,ti OR glott*:ab,ti OR supraglott*:ab,ti OR subglott*:ab,ti OR tongue:ab,ti OR pallat*:ab,ti) AND (neoplasm*:ab,ti OR ‘cancer’:ab,ti OR tumor*:ab,ti OR tumour*:ab,ti OR carcinoma*:ab,ti)))

**AND** (‘diffusion weighted imaging’/de OR ‘perfusion weighted imaging’/de OR ‘echo planar imaging’/de OR ‘nuclear magnetic resonance imaging’/de OR ‘MRI’:ab,ti OR ‘magnetic resonance imaging’:ab,ti OR ‘diffusion’:ab,ti OR ‘DWI’:ab,ti OR ‘perfusion’:ab,ti OR ‘dynamic contrast’:ab,ti OR ‘contrast enhanced’:ab,ti OR ‘dynamic susceptibility contrast’:ab,ti OR ‘DSC’:ab,ti)

**AND** (‘cancer recurrence’/de OR ‘recurrent disease’/de OR ‘minimal residual disease’/de OR ‘radiation necrosis’/de OR ‘cancer growth’/de OR ‘cancer regressive’/de OR ‘disease clearance’/de OR ‘relapse’/de OR ‘remission’/de OR ‘survival’/exp OR ‘terminal disease’/de OR ‘tumor growth’/de OR ‘tumor recurrence’/de OR ‘tumor regression’/de OR differentiat*:ab,ti OR ‘differential’:ab,ti OR discriminat*:ab,ti OR ‘treatment-induced’:ab,ti OR ‘posttreatment’:ab,ti OR ‘post treatment’:ab,ti OR ‘posttherapeutic’:ab,ti OR ‘post therapeutic’:ab,ti OR ‘post irradiation’:ab,ti OR ‘post radiation’:ab,ti OR ‘postradiation’:ab,ti OR ‘residual’:ab,ti OR ‘necrosis’:ab,ti OR ‘necrotic’:ab,ti OR ‘radiation injuries’:ab,ti)

**AND** (recurren*:ab,ti OR ‘true’:ab,ti OR 'response':ab,ti OR 'treatment outcome':ab,ti OR 'disease progression':ab,ti))

**Search strategy web of science:**

(TS=(“head and neck” OR "mouth" OR "Otorhinolaryngologic" OR “pharynx” OR “pharyngeal” OR “nasopharynx” OR “naspharyngeal” OR “oropharynx” OR “oropharyngeal” OR “hypopharynx” OR “hypopharyngeal” OR “oral cavity” OR “larynx” OR “laryngeal” OR “glottic” OR “glottal” OR “glottis” OR “supraglottic” OR “supraglottal” OR “supraglottis” OR “subglottic” OR “subglottal” OR “subglottis” OR “tongue” OR “palatal” OR “pallatum”) AND TS=(“neoplasma” OR “neoplasmas” OR “neoplasm” OR “cancer” OR “tumor” OR “tumour” OR “carcinoma” OR “tumours” OR “tumors” OR “malignancy” OR “malignancies”))

**AND** (TS=("magnetic resonance imaging" OR "MRI" OR "diffusion" OR "DWI” OR “perfusion” OR “dynamic contrast” OR “contrast enhanced” OR "dynamic susceptibility contrast" OR “DSC”))

**AND** (TS=(“differentiation” OR “differential” OR “discrimination” OR “treatment-induced” OR “posttreatment” OR “post treatment” OR “posttherapeutic” OR “post therapeutic” OR “post irradiation” OR “post radiation” OR “postradiation” OR “residual” OR “necrosis” OR “necrotic” OR “radiation injuries”) )

**AND (**TS=(“recurrence” OR “recurrent” OR “true” OR "response" OR "treatment outcome" OR "disease progression"))
