# Supplementary material for: Diagnostic accuracy of magnetic resonance imaging techniques for treatment response evaluation in patients with head and neck tumors, a systematic review and meta-analysis
Source: PLoS One. 2017 May 24;12(5):e0177986. doi: 10.1371/journal.pone.0177986 (PMC5443521; doi:10.1371/journal.pone.0177986)
Supplement: S1 Fig — Diagnostic accuracy and the 2x2 table is displayed with true positives (TP), false positives (FP), false negatives (FN) and true negative (TN). Sensitivity and specificity with the 95% Confidence intervals (CI) are given. (PDF) [file pone.0177986.s003.pdf]

SUPPLEMENTARY FIGURE 1 – Forest plots with diagnostic accuracy of anatomical MRI and ADC for primary tumor and nodal sites

A. Anatomical MRI primary site

Study	TP	FP	FN	TN	Sensitivity (95% CI)		Specificity (95% CI)	
Bhatia et al., 2010.[17]	11	4	8	38	58	(33-88)	90	(77-97)
Chan et al., 2006 group A.[18A]	21	3	1	9	95	(77-100)	75	(43-95)
Chan et al., 2006 group B.[18B]	3	11	1	197	75	(19-99)	95	(91-97)
Chong and Fan, 1997.[19]	5	7	4	29	56	(21-86)	81	(64-92)
Comoretto et al., 2008.[20]	27	4	1	31	96	(82-100)	89	(73-97)
King et al., 2013.[24]	9	7	0	21	100	(66-100)	75	(55-89)
Ljumanovic et al., 2008.[26]	25	13	1	41	96	(80-100)	76	(62-87)
Ng et al., 2010.[27]	25	7	4	143	86	(68-96)	95	(91-98)
Tshering Vogel et al., 2013.[28]	13	12	5	16	72	(47-90)	57	(37-76)
Vandecaveye et al., 2010.[29]	6	6	2	16	75	(35-97)	73	(50-89)
Yen et al., 2003.[31]	13	26	8	20	62	(38-82)	43	(29-59)

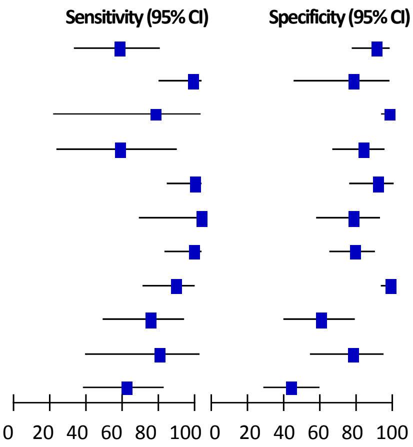

B. ADC primary site

Study	TP	FP	FN	TN	Sensitivity (95% CI)		Specificity (95% CI)	
Gouhar and El-Harir, 2011.[21]	11	1	2	7	85	(55-98)	88	(77-100)
Hong et al., 2013.[22]	16	40	7	71	70	(47-87)	64	(54-73)
Hwang et al., 2013.[23]	19	4	1	9	95	(75-100)	69	(39-91)
King et al., 2013.[25]	10	4	3	13	77	(46-95)	76	(50-93)
Tshering Vogel et al., 2013.[28]	17	0	1	21	94	(73-100)	100	(84-100)
Vandecaveye et al., 2010.[29]	8	1	0	21	100	(63-100)	95	(77-100)

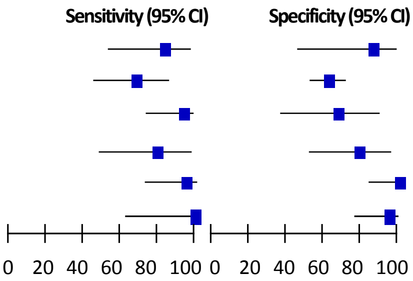

C. Anatomical MRI nodal site

Study	TP	FP	FN	TN	Sensitivity (95% CI)		Specificity (95% CI)	
Berrak et al., 2011.[16]	13	2	2	1	87	(60-98)	33	(1-91)
Comoretto et al., 2008.[20]	19	4	2	38	90	(70-99)	90	(77-97)
Ng et al., 2010.[27]	22	5	3	149	88	(69-97)	97	(93-99)
Vandecaveye et al., 2010.[29]	6	11	3	30	67	(30-93)	73	(57-86)

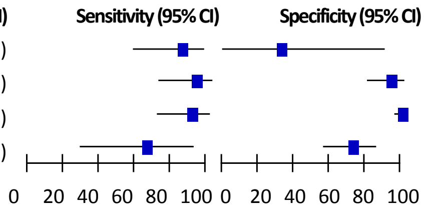

D. ADC nodal site

Study	TP	FP	FN	TN	Sensitivity (95% CI)		Specificity (95% CI)	
Berrak et al., 2011.[16]	11	0	4	3	73	(45-92)	100	(29-100)
Vandecaveye et al., 2010.[29]	7	5	2	36	78	(40-97)	88	(74-96)

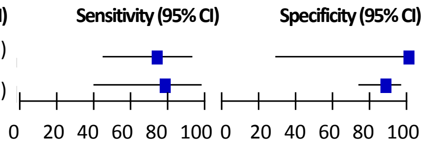

Diagnostic accuracy and the 2x2 table is displayed with true positives (TP), false positives (FP), false negatives (FN) and true negative (TN). Sensitivity and specificity with the 95% Confidence intervals (CI) are given.
