# Supplementary material for: Diagnostic accuracy of magnetic resonance imaging techniques for treatment response evaluation in patients with head and neck tumors, a systematic review and meta-analysis
Source: PLoS One. 2017 May 24;12(5):e0177986. doi: 10.1371/journal.pone.0177986 (PMC5443521; doi:10.1371/journal.pone.0177986)
Supplement: S2 Fig — See caption S1. (PDF) [file pone.0177986.s004.pdf]

SUPPLEMENTARY FIGURE 2 – Forest plots with diagnostic accuracy anatomical MRI and ADC for different scan times for the primary tumor site

A. Anatomical MRI primary site during treatment

Study	TP	FP	FN	TN	Sensitivity (95% CI)		Specificity (95% CI)	
Bhatia et al., 2010.[17]	13	8	8	23	76	(50-93)	74	(55-88)
Vandecaveye et al., 2010.[29]	7	10	2	13	88	(47-100)	57	(34-77)

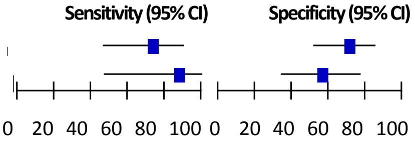

B. Anatomical MRI primary site early posttreatment

Study	TP	FP	FN	TN	Sensitivity (95% CI)		Specificity (95% CI)	
Bhatia et al., 2010.[17]	11	4	8	38	58	(33-80)	90	(77-97)
King et al., 2013.[24]	9	7	0	21	100	(66-100)	75	(55-89)
Vandecaveye et al., 2010.[29]	6	6	2	16	75	(35-97)	73	(50-89)

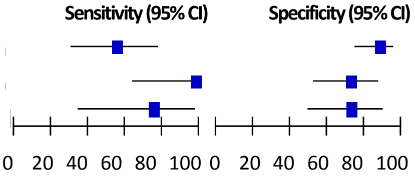

C. Anatomical MRI primary site late posttreatment

Study	TP	FP	FN	TN	Sensitivity (95% CI)		Specificity (95% CI)	
Chan et al., 2006 group A.[18A]	21	3	1	9	95	(77-100)	75	(43-95)
Chan et al., 2006 group B.[18B]	3	11	1	197	75	(19-99)	95	(91-97)
Chong and Fan, 1997.[19]	5	7	4	29	56	(21-86)	81	(64-92)
Comoretto et al., 2008.[20]	27	4	1	31	96	(82-100)	89	(73-97)
Ljumanovic et al., 2008.[26]	25	13	1	41	96	(80-100)	76	(62-87)
Ng et al., 2010.[27]	25	7	4	143	86	(68-96)	95	(91-98)
Tshering Vogel et al., 2013.[28]	13	12	5	16	72	(47-90)	57	(37-76)
Yen et al., 2003.[31]	13	26	8	20	62	(38-82)	43	(29-59)

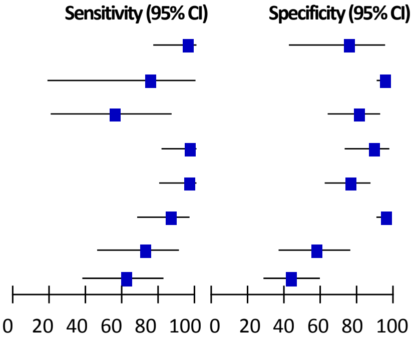

D. ADC primary site during treatment

Study	TP	FP	FN	TN	Sensitivity (95% CI)		Specificity (95% CI)	
Hong et al., 2013.[22]	16	40	7	71	70	(47-87)	64	(54-73)
King et al., 2013.[25]	10	4	3	13	77	(46-95)	76	(50-93)
Vandecaveye et al., 2010.[29]	8	5	2	39	80	(44-97)	89	(75-96)

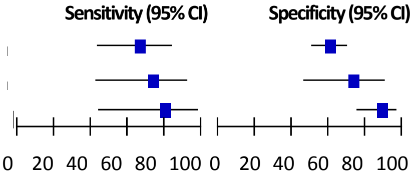

E. ADC primary site early posttreatment

Study	TP	FP	FN	TN	Sensitivity (95% CI)		Specificity (95% CI)	
Vandecaveye et al., 2010.[29]	8	1	0	21	100	(63-100)	95	(77-100)

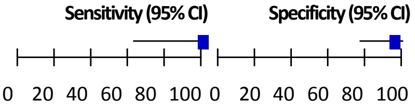

F. ADC primary site late posttreatment

Study	TP	FP	FN	TN	Sensitivity (95% CI)		Specificity (95% CI)	
Gouhar and El-Harir, 2011.[21]	11	1	2	7	85	(55-98)	88	(47-100)
Hwang et al., 2013.[23]	19	4	1	9	95	(75-100)	69	(39-91)
Tshering Vogel et al., 2010.[28]	17	0	1	21	94	(73-100)	100	(84-100)

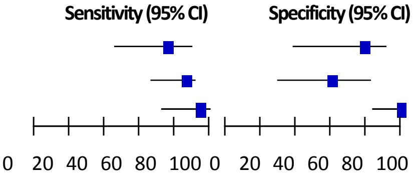

Diagnostic accuracy and the 2x2 table is displayed with true positives (TP), false positives (FP), false negatives (FN) and true negative (TN). Sensitivity and specificity with the 95% Confidence intervals (CI) are given.
